# Characteristics of lymphocyte subsets and cytokine profiles of patients with COVID-19

**DOI:** 10.1186/s12985-022-01786-2

**Published:** 2022-03-28

**Authors:** Pengfei Pan, Xinxin Du, Qilong Zhou, Yong Cui, Xiaochun Deng, Chao Liu, Zongjun Hu, Jianguo Chen, Xiangyou Yu, Weihua Shi

**Affiliations:** 1 Department of Critical Care Medicine, Chongqing University Three Gorges Hospital, Chongqing, 404100 China; 2 Department of Critical Care Medicine, The First Affiliated Hospital of Xinjiang Medical University, Ürümqi, 830054 China; 3 Department of Traditional Chinese Medicine, Chongqing University Three Gorges Hospital, Chongqing, 404100 China

**Keywords:** Coronavirus disease, Lymphocyte subsets, Cytokines

## Abstract

**Background:**

Abnormalities of lymphocyte subsets and cytokine profiles have been observed in most patients with coronavirus disease (COVID-19). Here, we explore the role of lymphocyte subsets and cytokines on hospital admission in predicting the severity of COVID-19.

**Methods:**

This study included 214 patients with COVID-19 who were treated at Chongqing University Three Gorges Hospital from January 19, 2020 to April 30, 2020. Any mutants were not detected in the studied patients. Patients were divided into non-intensive care unit (ICU) (mild/moderate) group and ICU (severe/critical) group, according to the severity of the disease. Clinical and laboratory data, including peripheral lymphocyte subsets and cytokines, were analyzed and compared. Logistic regression was used to analyze the predictive factors for ICU admission. Receiver operating characteristic (ROC) curves were drawn to evaluate the predictive value of selected indicators for the severity of COVID-19.

**Results:**

Of the 214 patients enrolled, 161 were non-ICU patients and 53 were ICU patients. Lymphopenia was observed in nearly all of ICU patients (96.2%) and 84.5% of non-ICU patients on hospital admission. The absolute number of lymphocytes, CD3^+^ T cells, CD4^+^ T cells, CD8^+^ T cells, CD19^+^ B cells, and natural killer (NK) cells were lower in ICU group (659.00 × 10^6^/L, 417.00 × 10^6^/L, 261.00 × 10^6^/L, 140.00 × 10^6^/L, 109.00 × 10^6^/L, 102.00 × 10^6^/L, respectively) than in non-ICU group (1063.00 × 10^9^/L, 717.00 × 10^6^/L, 432.00 × 10^6^/L, 271.00 × 10^6^/L, 133.00 × 10^6^/L, 143.00 × 10^6^/L, respectively). Interleukin (IL)-6 was significantly higher in ICU patients than in non-ICU patients (18.08 pg/mL vs. 3.13 pg/mL, *P* < 0.001). Multivariate logistic regression analysis showed that age (odds ratio: 1.067 [1.034–1.101]), diabetes mellitus (odds ratio: 9.154 [2.710–30.926]), CD3^+^ T cells (odds ratio: 0.996 [0.994–0.997]), and IL-6 (odds ratio: 1.006 [1.000–1.013]) were independent predictors for the development of severe disease. ROC curve analysis showed that the area under the ROC curve (AUC) of CD3^+^ T cells and IL-6 was 0.806 (0.737–0.874) and 0.785 (0.705–0.864), respectively, and the cutoff values were 510.50 × 10^6^/L (sensitivity, 71.7%; specificity, 79.5%) and 6.58 pg/mL (77.4%, 74.5%), respectively. There were no statistical differences among all tested indicators of lymphocyte subsets and cytokines between severe group (*n* = 38) and critical group (*n* = 15) on hospital admission or ICU admission, respectively.

**Conclusions:**

The levels of lymphocyte subsets decreased and the level of IL-6 increased significantly in ICU COVID-19 patients compared with non-ICU COVID-19 patients. Therefore, the number of CD3^+^ T cells and the level of IL-6 on hospital admission may serve as predictive factors for identifying patients with wild-type virus infection who will have severe disease.

## Introduction

The recent outbreak of severe acute respiratory syndrome-coronavirus 2 (SARS-CoV-2) has led to the declaration of a pandemic, which has seriously threatened human health and global public health security [1–4]. Initially, the lung was considered to be the organ most commonly damaged by SARS-CoV-2. However, the virus can also affect the nervous system, digestive system, urinary system, blood system, and other systems. On February 11, 2020, the World Health Organization declared the disease caused by SARS-CoV-2 as coronavirus disease (COVID-19). By June 20, 2021, more than 177 million individuals worldwide were infected [5]. Generally, most patients with COVID-19 did not become critically ill and recovered quickly; however, COVID-19 can also lead to death, with a case-fatality rate ranging from 0.7% to 10.8% [6–8]. It has been reported that the incubation period for COVID-19 is approximately 3–7 days. In the early stages, most patients have mild symptoms, but some of them develop acute respiratory distress syndrome, rapid acute respiratory failure, and even multiple organ failure. Therefore, preparing intensive care units (ICU) to respond to this crisis is of great importance. The Surviving Sepsis Campaign COVID-19 panel has issued several recommendations to guide clinical management of critically ill COVID-19 patients in ICU [9].

SARS-CoV-2 is a novel β-coronavirus that has at least 79.6% genetic sequence similarity with SARS-CoV, the virus responsible for the SARS outbreak in 2003 [10]. SARS-CoV-2 reaches the host cells by the angiotensin-converting enzyme II receptor, which is mainly expressed on pulmonary epithelial cells, but also on lymphocytes and other cell types [11]. Currently, immune disorders and cytokine storms contribute to the pathogenesis and progression of COVID-19. Significant lymphocytopenia has also been observed during the acute phase of COVID-19 [12]. In addition, an increasing number of studies have shown that changes in lymphocyte subsets, cytokines, and dysregulation of the host immune response in patients with different severities of COVID-19 [13–15]. A previous meta-analysis demonstrated that severe COVID-19 is closely associated with a decrease in lymphocytes and lymphocyte subsets, as well as the elevation of C-reactive protein (CRP), procalcitonin (PCT), and cytokines, but not interleukin (IL)-1β and IL-17 [16].

The aim of the current study was to describe the changes in peripheral blood lymphocyte subsets and cytokine profiles in patients with COVID-19, and to explore the role of these parameters on hospital admission in predicting the severity of COVID-19.

## Methods

### Study design and participants

This was a single-center, retrospective, cohort study. The study was approved by the Institutional Ethics Board of Chongqing University Three Gorges Hospital. Laboratory-confirmed patients with COVID-19 who were admitted to the hospital from January 19, 2020 to April 30, 2020 were enrolled in the study. Written informed consent was waived by the Ethics Board of the hospital for emerging infectious diseases, and oral consent was obtained from the patients.

Chongqing University Three Gorges Hospital, located in Wanzhou, Chongqing, is one of the major tertiary teaching hospitals and has been assigned by the government to be responsible for treating patients with COVID-19. All confirmed patients in the northeast area of Chongqing were admitted to our hospital. Any mutation analysis has not been performed, and it is assumed that all the studied patients were infected with SARS-CoV-2 wild-type (WT) strain.

In the study, patients who met any of the following criteria were excluded: (1) age < 18 years old, and (2) lack of complete cytokine data. The included patients were divided into four groups, according to the Novel Coronavirus Pneumonia Treatment Scheme issued by the National Health Commission of the People’s Republic of China (7th edition), on hospital admission or during hospitalization as follows [17]: (1) mild patients, in whom all of the following conditions were met: (i) epidemiological history, (ii) with mild clinical symptoms and normal imaging findings in both lungs, and (iii) positive result of reverse transcription-polymerase chain reaction (RT-PCR) for SARS-CoV-2 RNA; (2) moderate patients, in whom any of the following conditions were met: (i) epidemiological history, (ii) fever or other respiratory symptoms, (iii) typical CT image abnormalities of viral pneumonia, and (iv) positive result of RT-PCR for SARS-CoV-2 RNA; and (3) severe patients, in whom any of the following conditions were met in addition to (2), (i) shortness of breath with respiratory rate ≥ 30 times/min, (ii) resting oxygen saturation ≤ 93%, or (iii) oxygenation index (arterial oxygen tension/fractional inspired oxygen) ≤ 300 mmHg (1 mmHg = 0.133 kPa); (4) critical, in whom any of the following conditions were met in addition to (2) or (3), (i) need for mechanical ventilation due to respiratory failure, (ii) shock, or (iii) requiring ICU care, with simultaneous failure of other organs. All of the patients were divided into non-ICU (mild/moderate) group and ICU (severe/critical) group. ICU group was subdivided into direct-ICU group and late-ICU group, according to whether they were admitted directly to ICU on admission or later to ICU after admission.

### Data collection

Researchers responsible for data collection were trained before the study began so that they could correctly fill out the case report forms and reduce errors. Data including demographic characteristics (age and sex), baseline comorbidities, clinical symptoms, laboratory findings on hospital admission and/or on ICU admission, severity assessment, and outcomes were obtained from the electronic medical records. Two researchers collected data independently and checked each other’s forms for mistakes. Clinical outcomes were followed until June 30, 2020.

### Statistical analysis

Categorical variables were described as frequency rates and percentages in each category, and compared using χ^2^ test or Fisher’s exact test. Continuous variables were described as medians with interquartile range (IQR) values, and compared using the Mann–Whitney *U* test or Wilcoxon signed-rank test. For comparisons, a two-sided α value of < 0.05 was considered statistically significant. Multiple logistic regression analysis was used to screen independent risk factors associated with severe/critical condition. The diagnostic values of selected parameters on hospital admission for differentiating non-ICU and ICU cases were assessed by receiver operating characteristic (ROC) curves and the area under ROC curve (AUC). Cutoff values were identified following Youden’s index of the ROC curve. All statistical analyses were conducted using SPSS 23.0 and GraphPad Prism 8.0.

## Results

### Baseline characteristics of patients with COVID-19

Patients with confirmed COVID-19 (n = 248) were admitted to our hospital, 31 of whom were excluded due to lack of cytokine data; therefore, 214 patients were included in the final analysis. The median age of the included patients was 49.00 years (IQR 39.00–56.00), and 117 (54.7%) were men. Diabetes mellitus (24 [11.2%]), hypertension (16 [7.5%]), and underlying pulmonary diseases (10 [4.7%]) were the primary coexisting conditions, and cough (137 [64.0%]) was the most common symptom. Almost half of the patients had a fever (112 [52.3%]). Other symptoms included expectoration (60 [28.0%]), fatigue (49 [22.9%]), shortness of breath (41 [19.2%]), chills (28 [13.1%]), anorexia (27 [12.6%]), myalgia (26 [12.1%]), and other symptoms (Table 1).

**Table 1:** Comparison of baseline characteristics between non-ICU and ICU groups

Characteristic	Total (*n* = 214)	Non-ICU group (*n* = 161)	ICU group (*n* = 53)	*P* value
*Sociodemographic*				
Age, median (IQR), years	49.00 (39.00–56.00)	46.00 (37.50–54.00)	57.00 (50.50–75.50)	< 0.001
Male/female, n (%)	117 (54.7)/97 (45.3)	88 (54.7)/73 (45.3)	29 (54.7)/24 (45.3)	0.994
*Chronic medical diseases*				
Hypertension, n (%)	16 (7.5)	11 (6.8)	5 (9.4)	0.746
Coronary heart disease, n (%)	7 (3.3)	3 (1.9)	4 (7.5)	0.116
Underlying pulmonary diseases, n (%)	10 (4.7)	4 (2.5)	6 (11.3)	0.023
Chronic liver or kidney disease, n (%)	5 (2.3)	3 (1.9)	2 (3.8)	0.599
Diabetes mellitus, n (%)	24 (11.2)	8 (5.0)	16 (30.2)	< 0.001
Malignancy, n (%)	2 (0.9)	0 (0.0)	2 (3.8)	0.060
*Signs and symptoms on hospital admission*				
Fever, n (%)	112 (52.3)	79 (49.1)	33 (62.3)	0.095
Chills, n (%)	28 (13.1)	19 (11.8)	9 (17.0)	0.332
Myalgia, n (%)	26 (12.1)	17 (10.6)	9 (17.0)	0.215
Fatigue, n (%)	49 (22.9)	29 (18.0)	20 (37.7)	0.003
Cough, n (%)	137 (64.0)	95 (59.0)	42 (79.2)	0.008
Expectoration, n (%)	60 (28.0)	39 (24.2)	21 (39.6)	0.030
Short of breath, n (%)	41 (19.2)	20 (12.4)	21 (39.6)	< 0.001
Chest tightness, n (%)	19 (8.9)	11 (6.8)	8 (15.1)	0.120
Chest pain, n (%)	6 (2.8)	2 (1.2)	4 (7.5)	0.034
Anorexia, n (%)	27 (12.6)	18 (11.2)	9 (17.0)	0.270
Nausea or vomiting, n (%)	9 (4.2)	6 (3.7)	3 (5.7)	0.831
Abdominal pain, n (%)	4 (1.9)	2 (1.2)	2 (3.8)	0.257
Diarrhea, n (%)	13 (6.1)	10 (6.2)	3 (5.7)	1.000
Headache, n (%)	21 (9.8)	15 (9.3)	6 (11.3)	0.671
Dizziness, n (%)	21 (9.8)	14 (8.7)	7 (13.2)	0.338
*Others*				
Onset of symptom to hospital admission, median (IQR), days	6.00 (3.00–10.00)	6.00 (3.00–10.00)	7.00 (4.50–8.00)	0.612
NCT of SARC-CoV-2, median (IQR), days	17.00 (13.00–23.00)	17.00 (13.00–23.00)	21.00 (15.50–26.50)	0.004
Length of hospital stay, median (IQR), days	14.00 (12.00–22.00)	13.00 (11.00–17.00)	24.00 (14.50–37.00)	< 0.001
Onset of symptom to hospital discharge or death, median (IQR), days	22.00 (18.00–29.00)	22.00 (18.00–29.00)	30.00 (22.50–43.50)	< 0.001

*IQR* interquartile range, *NCT* negative conversion time

Of these 214 patients, 1 (0.5%) had mild disease, 160 (74.8%) had moderate disease, 37 (17.3%) had severe disease, and 16 (7.5%) had critical disease. ICU patients (53 [24.8%]) were significantly older than non-ICU patients (161 [75.2%]; 57.00 years [50.50–75.50] vs. 46.00 years [IQR 37.50–54.00], *P* < 0.001). There were more patients with diabetes mellitus in ICU group than in non-ICU group (16 [30.2%] vs. 8 [5.0%], *P* < 0.001) and underlying pulmonary diseases (6 [11.3%] vs. 4 [2.5%], *P* < *0.01*. 023). There were no significant differences between the two cohorts with regard to hypertension and coronary heart disease. Compared with non-ICU patients, ICU patients tended to report fatigue, cough, expectoration, shortness of breath, and chest pain (Table 1).

### Laboratory findings of patients with COVID-19 on hospital admission

There were obvious differences in the laboratory findings between ICU and non-ICU group patients. No abnormalities in white blood cell counts were observed in most patients. Neutrophil counts were significantly higher in ICU patients than in non-ICU patients (4.21 × 10^9^/L [IQR 2.97–5.66] vs. 3.46 × 10^9^/L [IQR 2.51–4.40], *P* = 0.006). Most patients with COVID-19 had a decrease in lymphocyte levels (187 [87.4%]). Lymphocyte counts decreased more significantly in ICU group than in non-ICU group (0.75 × 10^9^/L [IQR 0.55–1.04] vs. 1.20 × 10^9^/L [IQR 0.90–1.60], *P* < 0.001). More patients in ICU group had thrombocytopenia than in non-ICU group (28.3% vs. 13.0%, *P* = 0.011). ICU patients had higher direct bilirubin (*P* = 0.033), aspartate aminotransferase (AST) (*P* < 0.001), activated partial thromboplastin time (APTT) (*P* < 0.001), prothrombin time (PT) (*P* = 0.019), and D-dimer (*P* < 0.001) than non-ICU patients on hospital admission. C-reactive protein (CRP) was significantly higher in ICU group than in non-ICU group (81.05 mg/L [IQR 46.12–131.49] vs. 5.99 mg/L [IQR 1.72–20.43], *P* < 0.001). In most patients, the procalcitonin (PCT) level was in the normal range (Table 2).

**Table 2:** Comparison of laboratory findings between non-ICU and ICU groups on hospital admission

Characteristic	Normal range	Total (*n* = 214)	Non-ICU group (*n* = 161)	ICU group (*n* = 53)	*P* value
*Blood routine*					
White blood cell, median (IQR), × 10^9^/L	3.50–9.50	5.20 (4.10–6.70)	5.10 (4.10–6.60)	5.40 (4.35–6.95)	0.490
Neutrophil, median (IQR), × 10^9^/L	1.80–6.30	3.56 (2.58–4.88)	3.46 (2.51–4.40)	4.21 (2.97–5.66)	0.006
Lymphocyte, median (IQR), × 10^9^/L	1.10–3.20	1.08 (0.80–1.53)	1.20 (0.90–1.60)	0.75 (0.55–1.04)	< 0.001
Monocyte, median (IQR), × 10^9^/L	3.00–10.00	0.37 (0.29–0.48)	0.39 (0.31–0.48)	0.33 (0.19–0.48)	0.016
Platelet, median (IQR), × 10^9^/L	125.00–350.00	176.50 (138.00–236.25)	186.00 (146.50–238.00)	150.00 (118.00–229.50)	0.045
Neutrophil, median (IQR), %	40.00–75.00	69.15 (60.68–78.75)	65.80 (58.70–73.45))	79.90 (70.05–84.90)	< 0.001
Lymphocyte, median (IQR), %	20.00–50.00	22.65 (14.30–29.20)	24.80 (17.95–30.65)	13.50 (10.05–21.50)	< 0.001
Monocyte, median (IQR),%	3.00–10.00	7.10 (5.50–9.20)	7.30 (6.00–9.25)	5.70 (3.90–8.45)	0.001
NLR, median (IQR), %	NA	3.05 (2.07–5.34)	2.55 (1.85–4.09)	5.94 (3.26–8.50)	< 0.001
Lymphocytopenia, n (%)	NA	187 (87.4)	136 (84.5)	51 (96.2)	0.025
Thrombopenia, n (%)	NA	36 (16.8)	21 (13.0)	15 (28.3)	0.011
*Blood biochemistry*					
Urea nitrogen, median (IQR), mmol/L	3.10–8.00	4.00 (3.20–5.20)	3.90 (3.20–4.95)	4.50 (3.05–5.90)	0.160
Creatinine, median (IQR), mmol/L	57.00–97.00	66.00 (55.00–75.25)	66.00 (56.00–76.50)	65.00 (50.00–75.00)	0.435
TBil, median (IQR), umol/L	0.00–26.00	9.60 (6.38–15.93)	9.60 (5.95–16.05)	10.00 (6.80–15.20)	0.768
DBil, median (IQR), umol/L	0.00–8.00	4.70 (3.28–6.40)	4.50 (3.10–6.20)	5.10 (3.65–8.15)	0.033
ALT, median (IQR), U/L	9.00–50.00	21.20 (14.68–35.13)	19.70 (14.65–33.35)	26.00 (13.55–39.30)	0.218
AST, median (IQR), U/L	15.00–40.00	23.50 (17.18–34.18)	20.80 (16.50–28.70)	34.00 (25.60–44.50)	< 0.001
CK, median (IQR), U/L	50.00–310.00	62.00 (42.00–90.00)	60.00 (42.00–84.05)	77.00 (40.50–159.50)	0.067
CKMB, median (IQR), U/L	0.00–25.00	12.00 (10.00–16.00)	12.00 (10.00–16.00)	14.00 (9.90–17.00)	0.530
HbA1c, median (IQR),%		5.60 (5.30–5.90)	5.50 (5.30–5.80)	5.90 (5.60–7.15)	< 0.001
*Infection related parameters*					
CRP, median (IQR), mg/L	0.00–8.00	11.63 (2.12–50.89)	5.99 (1.72–20.43)	81.05 (46.12–131.49)	< 0.001
PCT, median (IQR), ng/mL	< 0.046	0.04 (0.03–0.07)	0.04 (0.03–0.06)	0.09 (0.06–0.14)	< 0.001
*Coagulation function*					
PT, median (IQR), s	8.00–14.00	11.10 (10.60–11.43)	11.00 (10.55–11.35)	11.30 (10.65–11.80)	0.019
APTT, median (IQR), s	20.00–40.00	26.50 (24.50–29.30)	26.10 (23.90–28.40)	29.00 (26.30–33.50)	< 0.001
D-dimer, median (IQR), mg/L	0.00–0.55	0.37 (0.20–0.60)	0.28 (0.19–0.47)	0.62 (0.44–1.30)	< 0.001

*ICU* intensive care unit, *NLR* neutrophil-to-lymphocyte ratio, *IQR* interquartile range, *TBil* total bilirubin, *DBil* direct bilirubin, *ALT* alanine aminotransferase, *AST* aspartate aminotransferase, *CK* creatine kinase, *CKMB* MB isoenzyme of creatine kinase, *HbA1c* glycated hemoglobin A1c, *CRP* C-reactive protein, *PCT* procalcitonin, *PT* prothrombin time, *APTT* activated partial thromboplastin time

### Lymphocyte subsets and cytokines of patients with COVID-19 on hospital admission

**Fig. 1:**
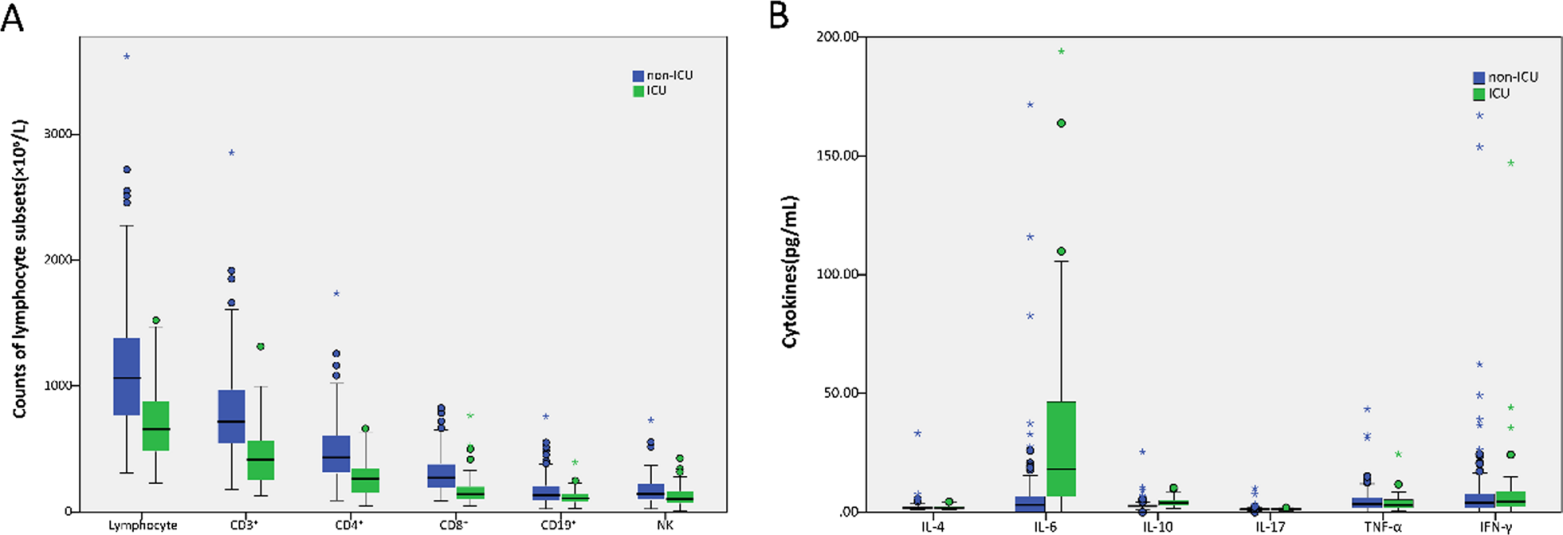
Comparison of lymphocyte subsets and cytokine profiles between ICU and non-ICU groups. **A** Counts of lymphocyte subsets. **B** Cytokines. *NK* natural killer, *IL* interleukin, *TNF* tumor necrosis factor, *IFN* interferon

The lymphocyte, CD3^+^ T cell, CD4^+^ T cell, CD8^+^ T cell, CD19^+^ B cell (total B cell), and natural killer (NK) cell counts were lower in ICU group (659.00 × 10^6^/L, 417.00 × 10^6^/L, 261.00 × 10^6^/L, 140.00 × 10^6^/L, 109.00 × 10^6^/L, 102.00 × 10^6^/L, respectively) than in non-ICU group (1063.00 × 10^9^/L, 717.00 × 10^6^/L, 432.00 × 10^6^/L, 271.00 × 10^6^/L, 133.00 × 10^6^/L, 143.00 × 10^6^/L, respectively). Interleukin (IL)-6 was significantly higher in ICU group than in non-ICU group (18.08 pg/mL vs. 3.13 pg/mL, *P* < 0.001). IL-10 was higher in ICU group than in non-ICU group (3.83 pg/mL vs. 2.56 pg/mL, *P* < 0.001), but still in the normal range. There was no significant difference between the two cohorts in terms of the CD4^+^/CD8^+^ ratio, IL-4, IL-17, tumor necrosis factor (TNF)-α, and interferon (IFN)-γ (Table 3, Fig. 1).

**Table 3:** Comparison of lymphocyte subsets and cytokines between non-ICU and ICU groups on hospital admission

Characteristic	Normal range	Total (*n* = 214)	Non- ICU group (*n* = 161)	ICU group (*n* = 53)	*P* value
*Lymphocyte classification*					
Lymphocyte, median (IQR), × 10^6^/L	1530.00–3700.00	936.50 (661.50–1323.25)	1063.00 (767.50–1389.50)	659.00 (480.50–885.50)	< 0.001
CD3^+^, median (IQR), × 10^6^/L	699.00–2540.00	632.50 (429.50–925.50)	717.00 (544.00–973.00)	417.00 (252.50–571.50)	< 0.001
CD4^+^, median (IQR), × 10^6^/L	410.00–1590.00	377.50 (265.75–536.00)	432.00 (308.50–609.50)	261.00 (153.50–351.50)	< 0.001
CD8^+^, median (IQR), × 10^6^/L	190.00–1140.00	242.50 (157.75–335.50)	271.00 (193.00–382.50)	140.00 (95.00–205.00)	< 0.001
CD19^+^, median (IQR), × 10^6^/L	90.00–660.00	128.00 (86.75–184.75)	133.00 (90.50–209.00)	109.00 (77.50–144.50)	0.012
NK, median (IQR), × 10^6^/L	90.00–590.00	132.00 (86.75–211.00)	143.00 (96.50–230.50)	102.00 (65.50–166.00)	0.002
CD3^+^, median (IQR),%	55.00–84.00	68.09 (61.74–75.00)	70.26 (64.23–75.48)	62.59 (54.61–68.75)	< 0.001
CD4^+^, median (IQR),%	31.00–60.00	39.30 (34.58–44.40)	40.05 (35.87–44.93)	37.78 (29.18–43.56)	0.030
CD8^+^, median (IQR),%	13.00–41.00	25.02 (20.28–30.41)	25.96 (21.03–30.75)	22.42 (17.52–29.01)	0.009
CD19^+^, median (IQR),%	6.00–25.00	14.54 (10.46–18.09)	13.94 (9.99–17.14)	17.48 (13.29–22.21)	< 0.001
NK, median (IQR),%	5.00–27.00	15.00 (9.59–21.97)	13.98 (9.11–20.85)	18.64 (10.10–25.99)	0.038
CD4^+^/CD8^+^, median (IQR)	0.70–2.87	1.54 (1.20–2.05)	1.54 (1.24–1.97)	1.53 (1.12–2.43)	0.649
*Cytokine profiles*					
IL-4, median (IQR), pg/mL	0.00–8.56	1.70 (1.33–2.38)	1.70 (1.34–2.37)	1.68 (1.26–2.45)	0.573
IL-6, median (IQR), pg/mL	0.00–5.40	4.15 (0.00–11.75)	3.13 (0.00–6.69)	18.08 (6.69–46.45)	< 0.001
IL-10, median (IQR), pg/mL	0.00–12.90	2.72 (2.33–3.76)	2.56 (2.23–3.08)	3.83 (2.98–5.16)	< 0.001
IL-17, median (IQR), pg/mL	0.00–21.40	1.21 (1.04–1.38)	1.21 (1.04–1.39)	1.18 (1.02–1.37)	0.393
TNF-α, median (IQR), pg/mL	0.00–16.50	3.39 (1.82–5.93)	3.48 (1.86–6.25)	3.05 (1.61–5.42)	0.390
IFN-γ, median (IQR), pg/mL	0.00–23.10	4.15 (1.77–8.28)	4.05 (1.70–7.88)	4.32 (2.32–8.72)	0.295

*ICU* intensive care unit, *IQR* interquartile range, *NK* natural killer, *IL* interleukin, *TNF* tumor necrosis factor, *IFN* interferon

### Multivariate logistic regression model and ROC curve analysis for ICU admission

Twelve univariate variables, including age, sex, underlying pulmonary diseases, diabetes mellitus, the counts of all tested lymphocyte subsets, and IL-6, were selected to perform multivariate logistic regression analysis to identify independent predictors of ICU admission. Stepwise forward method was used. The results showed that age (*OR* 1.067; *95% CI* 1.034–1.101; *P* < 0.001), diabetes mellitus (*OR* 9.154; *95% CI* 2.710–30.926; *P* < 0.001), CD3^+^ T cell counts (*OR* 0.996; *95% CI* 0.994–0.997; *P* < 0.001), and IL–6 (*OR* 1.006; *95% CI* 1.000–1.013; *P* = 0.039) on hospital admission were risk factors of ICU cases with COVID-19. The following equation was obtained: Probability (severe/critical COVID-19) = 1/1 + exp − [− 2.458 + (0.065 × age) + 2.214 × diabetes mellitus + (− 0.004 × CD3^+^ T cell count) + (0.006 × IL–6) (Table 4).

**Table 4:** Multivariate logistic regression model of predictors for ICU admission

Variables	β	*OR*	*95% CI*	*P* value
Age (years)	0.065	1.067	1.034–1.101	< 0.001
Diabetes mellitus (1 = yes, 0 = no)	2.214	9.154	2.710–30.926	< 0.001
CD3^+^ (× 10^6^/L)	− 0.004	0.996	0.994–0.997	< 0.001
IL-6 (pg/ml)		1.006	1.000–1.013	0.039
Constant	− 2.458	0.086	–	0.014

*ICU* intensive care unit, *IL* interleukin

**Fig. 2:**
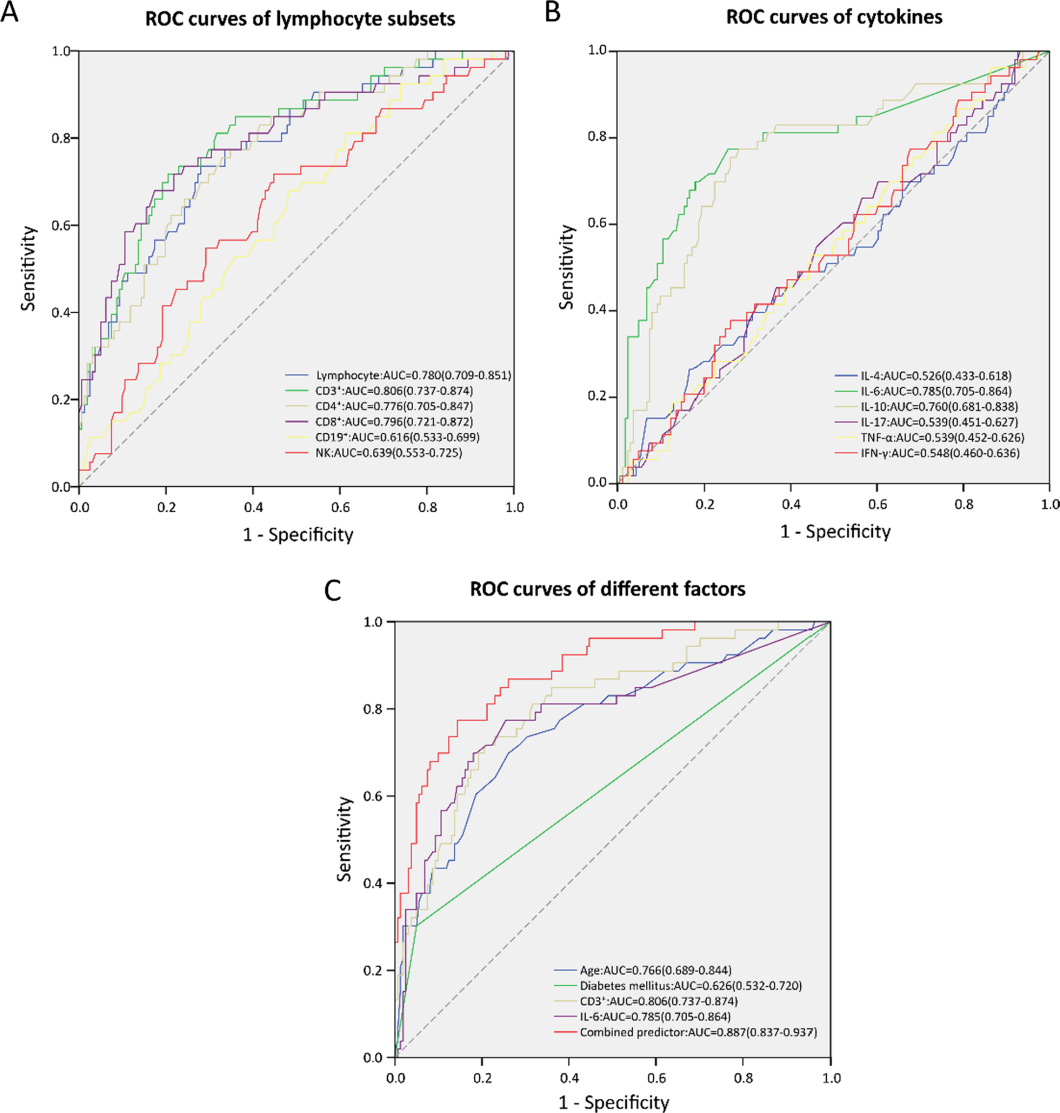
ROC curve analysis of different predictors for ICU admission. **A** ROC curves of lymphocyte subsets. **B** ROC curves of lymphocyte cytokines. **C** ROC curves of different factors. *ROC* receiver operating characteristic, *AUC* area under ROC curve, *NK* natural killer, *IL* interleukin, *TNF* tumor necrosis factor, *IFN* interferon

Next, ROC curve analysis was performed to further evaluate the predictive accuracy of different variables. The results showed that the AUC of the CD3^+^ T cell count and IL-6 on hospital admission were 0.806 (*95% CI* 0.737–0.874; *P* < 0.001) and 0.785 (*95% CI* 0.705–0.864; *P* < 0.001); the cutoff values were 357.50 × 10^6^/L (sensitivity, 77.4%; specificity, 65.2%) and 6.58 pg/mL (77.4%, 74.5%), respectively. Moreover, the ROC curve of the model, combining age, diabetes mellitus, CD3^+^ T cell count reduction, and IL–6 elevation, had a larger AUC (0.887 [*95% CI* 0.837–0.937; *P* < 0.001) with the cutoff value of 0.309 (sensitivity 77.4%, specificity 85.7%) (Table 5, Fig. 2).

**Table 5:** Summary of ROC curve parameters for predicting ICU admission

Predictor	AUC	*P* value	95% *CI*	Cutoff value	Jordan index	Sensitivity (%)	Specificity
Age	0.766	< 0.001	0.689–0.844	> 53.50	0.437	69.8	73.9
Diabetes mellitus	0.626	0.006	0.532–0.720	> 0.50	0.252	30.2	95.0
Lymphocyte	0.780	< 0.001	0.709–0.851	< 840.00	0.456	73.6	72.0
CD3^+^	0.806	< 0.001	0.737–0.874	< 510.50	0.512	71.7	79.5
CD4^+^	0.776	< 0.001	0.705–0.847	< 357.50	0.426	77.4	65.2
CD8^+^	0.796	< 0.001	0.721–0.872	< 173.50	0.505	67.9	82.6
CD19^+^	0.616	0.012	0.533–0.699	< 148.00	0.196	81.1	38.5
NK	0.639	0.002	0.553–0.725	< 134.50	0.270	71.7	55.3
IL-4	0.526	0.573	0.433–0.618	< 1.27	0.096	26.4	83.2
IL-6	0.785	< 0.001	0.705–0.864	> 6.58	0.519	77.4	74.5
IL-10	0.760	< 0.001	0.681–0.838	> 2.95	0.494	77.4	72.0
IL-17	0.539	0.393	0.451–0.627	< 1.27	0.096	69.8	39.8
TNF-α	0.539	0.390	0.452–0.626	< 8.40	0.099	96.2	13.7
IFN-γ	0.548	0.295	0.460–0.636	> 7.73	0.116	37.7	73.9
Combined predictor	0.887	< 0.001	0.837–0.937	> 0.309	0.631	77.4	85.7

*ROC* receiver operating characteristic, *ICU* intensive care unit, *NK* natural killer, *IL* interleukin, *TNF* tumor necrosis factor, *IFN* interferon

### Lymphocyte subsets and cytokines of ICU COVID-19 patients

Of the 53 patients in ICU group, there were no statistical differences among all tested indicators of lymphocyte subsets and cytokines between severe group (*n* = 37) and critical group (*n* = 16) on hospital or ICU admission (Tables 6, 7).

**Table 6:** Comparison of lymphocyte subsets and cytokines between severe and critical groups on hospital admission

Characteristic	Total (*n* = 53)	Severe group (*n* = 37)	Critical group (*n* = 16)	*Z* value	*P* value
*Lymphocyte classification*					
Lymphocyte, median (IQR), × 10^6/^L	659.00 (480.50–885.50)	743.00 (517.00–965.50)	547.00 (473.00–704.75)	− 1.792	0.073
CD3^+^, median (IQR), × 10^6/^L	417.00 (252.50–571.50)	430.00 (327.00–602.00)	327.00 (211.75–476.75)	− 1.482	0.138
CD4^+^, median (IQR), × 10^6^/L	261.00 (153.50–351.50)	280.00 (173.00–380.50)	211.00 (131.50–294.75)	− 1.782	0.075
CD8^+^, median (IQR), × 10^6^/L	140.00 (95.00–205.00)	148.00 (95.00–234.50)	120.00 (90.75–160.25)	− 1.298	0.194
CD19^+^, median (IQR), × 10^6^/L	109.00 (77.50–144.50)	116.00 (86.00–146.00)	99.00 (46.00–128.75)	− 1.579	0.114
NK, median (IQR), × 10^6^/L	102.00 (65.50–166.00)	103.00 (77.50–175.00)	87.00 (49.50–133.50)	− 1.298	0.194
CD3^+^, median (IQR),%	62.59 (54.61–68.75)	62.59 (56.71–68.28)	61.37 (49.94–70.59)	− 0.019	0.985
CD4^+^, median (IQR),%	37.78 (29.18–43.56)	38.54 (30.89–43.93)	30.14 (26.93–40.06)	− 1.376	0.169
CD8^+^, median (IQR),%	22.42 (17.52–29.01)	21.13 (17.32–27.49)	22.86 (18.58–36.27)	0.872	0.383
CD19^+^, median (IQR),%	17.48 (13.29–22.21)	17.00 (14.65–21.74)	18.87 (11.86–22.82)	− 0.194	0.846
NK, median (IQR),%	18.64 (10.10–25.99)	16.68 (12.29–25.77)	19.39 (9.23–26.18)	0.039	0.969
CD4^+^/CD8^+^, median (IQR)	1.53 (1.12–2.43)	1.82 (1.20–2.34)	1.24 (0.84–2.55)	− 1.066	0.287
*Cytokine profiles*					
IL-4, median (IQR), pg/mL	1.68 (1.26–2.45)	1.55 (1.20–2.38)	1.99 (1.32–2.50)	1.027	0.304
IL-6, median (IQR), pg/mL	18.08 (6.69–46.45)	17.35 (6.69–43.62)	21.89 (7.20–85.51)	1.077	0.281
IL-10, median (IQR), pg/mL	3.83 (2.98–5.16)	3.55 (2.81–4.94)	4.79 (3.23–6.25)	1.705	0.088
IL-17, median (IQR), pg/mL	1.18 (1.02–1.37)	1.15 (1.00–1.33)	1.20 (1.04–1.47)	0.349	0.727
TNF-α, median (IQR), pg/mL	3.05 (1.61–5.42)	3.12 (1.36–5.59)	2.93 (2.19–5.25)	0.281	0.779
IFN-γ, median (IQR), pg/mL	4.32 (2.32–8.72)	5.18 (2.00–10.04)	4.08 (2.35–7.44)	− 0.562	0.574

*NK* natural killer, *IL* interleukin, *TNF* tumor necrosis factor, *IFN* interferon

**Table 7:** Comparison of lymphocyte subsets and cytokines between severe and critical groups on ICU admission

Characteristic	Total (*n* = 53)	Severe group (*n* = 37)	Critical group (*n* = 16)	*Z* value	*P* value
*Lymphocyte classification*					
Lymphocyte, median (IQR), × 10^6/^L	597.00 (420.50–870.00)	695.00 (423.00–902.00)	535.00 (367.50–704.75)	− 1.395	0.163
CD3^+^, median (IQR), × 10^6/^L	399.00 (196.50–547.50)	430.00 (211.00–590.00)	327.00 (175.25–476.75)	− 1.085	0.278
CD4^+^, median (IQR), × 10^6^/L	243.00 (118.00–341.00)	261.00 (131.50–377.00)	211.00 (86.50–294.75)	− 1.279	0.201
CD8^+^, median (IQR), × 10^6^/L	140.00 (87.50–209.00)	157.00 (89.00–227.50)	117.50 (84.75–160.25)	− 1.143	0.253
CD19^+^, median (IQR), × 10^6^/L	103.00 (73.50–144.50)	103.00 (80.00–146.00)	99.00 (46.00–131.00)	− 1.163	0.245
NK, median (IQR), × 10^6^/L	103.00 (60.50–166.00)	109.00 (67.50–168.50)	87.00 (49.50–130.00)	− 1.424	0.154
CD3^+^, median (IQR),%	61.33 (50.67–68.25)	61.33 (50.96–67.90)	61.37 (49.94–70.59)	0.446	0.656
CD4^+^, median (IQR),%	35.84 (28.26–42.69)	36.04 (29.87–42.89)	30.14 (26.93–40.06)	− 0.911	0.363
CD8^+^, median (IQR),%	21.98 (16.61–28.62)	21.10 (16.61–27.49)	22.77 (16.09–36.27)	0.581	0.561
CD19^+^, median (IQR),%	17.92 (13.04–22.91)	17.73 (14.38–22.93)	18.87 (11.86–22.82)	− 0.271	0.786
NK, median (IQR),%	19.28 (10.66–26.77)	19.18 (13.41–27.32)	19.39 (9.23–26.18)	− 0.542	0.587
CD4^+^/CD8^+^, median (IQR)	1.53 (1.09–2.33)	1.71 (1.12–2.08)	1.23 (0.83–2.55)	0.794	0.416
*Cytokine profiles*					
IL-4, median (IQR), pg/mL	1.73 (1.33–2.45)	1.68 (1.32–2.45)	1.98 (1.32–2.50)	0.523	0.607
IL-6, median (IQR), pg/mL	16.39 (4.53–49.63)	11.61 (2.48–46.45)	21.89 (7.20–85.51)	1.437	0.151
IL-10, median (IQR), pg/mL	3.76 (2.85–5.17)	3.44 (2.70–5.17)	4.79 (3.23–6.25)	1.744	0.081
IL-17, median (IQR), pg/mL	1.19 (1.04–1.40)	1.18 (1.04–1.36)	1.20 (1.04–1.47)	− 0.058	0.954
TNF-α, median (IQR), pg/mL	3.52 (2.14–5.42)	3.54 (1.92–5.59)	2.93 (2.19–5.25)	− 0.203	0.839
IFN-γ, median (IQR), pg/mL	5.18 (2.27–8.72)	5.42 (1.95–9.81)	4.08 (2.35–7.44)	− 0.668	0.504

*ICU* intensive care unit, *NK* natural killer, *IL* interleukin, *TNF* tumor necrosis factor, *IFN* interferon

Moreover, among the patients in ICU group, 33 patients (62.3%) were admitted directly to ICU, and 20 (37.7%) had delayed admission to ICU by 2 days from the point of hospital admission (IQR 1–4). There were no statistical differences among all tested indicators of lymphocyte subsets and cytokines between direct-ICU group and late-ICU group on hospital admission (Table 8).

**Table 8:** Comparison of lymphocyte subsets and cytokines between direct-ICU and late-ICU groups on hospital admission

Characteristic	Direct-ICU group (*n* = 33)	Late-ICU group (*n* = 20)	*Z* value	*P* value
*Lymphocyte classification*				
Lymphocyte, median (IQR), × 10^6/^L	681.00 (480.50–965.50)	578.50 (489.50–820.25)	− 0.706	0.480
CD3^+^, median (IQR), × 10^6/^L	430.00 (220.50–571.50)	357.00 (264.00–573.50)	− 0.505	0.614
CD4^+^, median (IQR), × 10^6^/L	282.00 (145.00–350.50)	236.00 (154.50–367.75)	− 0.422	0.673
CD8^+^, median (IQR), × 10^6^/L	145.00 (95.00–225.50)	135.00 (90.75–185.00)	− 0.340	0.734
CD19^+^, median (IQR), × 10^6^/L	119.00 (77.50–156.50)	102.00 (72.25–130.75)	− 1.000	0.317
NK, median (IQR), × 10^6^/L	86.00 (60.00–175.00)	103.00 (82.25–124.75)	0.284	0.776
CD3^+^, median (IQR),%	61.22 (53.58–67.36)	66.93 (55.12–69.16)	1.229	0.219
CD4^+^, median (IQR),%	37.78 (29.39–42.93)	38.36 (27.61–46.54)	0.440	0.660
CD8^+^, median (IQR),%	20.60 (17.11–28.12)	23.09 (17.65–29.87)	0.706	0.480
CD19^+^, median (IQR),%	17.98 (15.78–22.67)	15.35 (11.19–20.80)	− 1.596	0.110
NK, median (IQR),%	16.68 (9.62–26.46)	18.95 (12.06–22.85)	0.037	0.971
CD4^+^/CD8^+^, median (IQR)	1.53 (1.15–2.43)	1.55 (1.10–2.51)	− 0.303	0.762
*Cytokine profiles*				
IL-4, median (IQR), pg/mL	1.68 (1.31–2.47)	1.64 (1.20–2.36)	− 0.725	0.468
IL-6, median (IQR), pg/mL	10.12 (2.52–43.99)	20.48 (14.27–51.22)	1.517	0.129
IL-10, median (IQR), pg/mL	3.76 (2.79–5.17)	3.85 (3.09–4.90)	0.339	0.734
IL-17, median (IQR), pg/mL	1.15 (1.04–1.36)	1.21 (0.96–1.43)	0.073	0.941
TNF-α, median (IQR), pg/mL	3.12 (1.88–5.04)	2.97 (1.30–5.84)	− 0.028	0.978
IFN-γ, median (IQR), pg/mL	5.18 (1.77–8.72)	3.73 (2.61–8.73)	0.009	0.993

*ICU* intensive care unit, *NK* natural killer, *IL* interleukin, *TNF* tumor necrosis factor, *IFN* interferon

Of the 20 late-ICU patients, 14 patients (70.0%) had delayed admission to ICU by > 2 days from hospital admission; all of them had their lymphocyte subsets and cytokines measured on hospital admission and on ICU admission. There were no statistical differences among most of the tested indicators of lymphocyte subsets and cytokines from hospital admission to ICU admission, but the lymphocyte, CD3^+^ T cell, CD4^+^ T cell, CD8^+^ T cell, and CD19^+^ B cell counts showed downward trends (Table 9).

**Table 9:** Comparison of lymphocyte subsets and cytokines of late-ICU group from hospital admission to ICU admission (*n* = 14)

Characteristic	Hospital admission	ICU admission	*Z* value	*P* value
*Lymphocyte classification*				
Lymphocyte, median (IQR), × 10^6/^L	561.00 (477.00–767.00)	498.50 (301.00–712.50)	− 1.682	0.093
CD3^+^, median (IQR), × 10^6/^L	347.00 (254.25–520.50)	267.00 (117.50–499.25)	− 1.784	0.074
CD4^+^, median (IQR), × 10^6^/L	236.00 (152.25–369.25)	174.00 (56.25–273.50)	− 1.784	0.074
CD8^+^, median (IQR), × 10^6^/L	125.00 (86.75–185.00)	101.00 (51.25–190.75)	− 1.580	0.114
CD19^+^, median (IQR), × 10^6^/L	108.50 (85.75–136.00)	98.50 (58.50–122.50)	− 1.887	0.059
NK, median (IQR), × 10^6^/L	99.50 (77.25–124.25)	97.00 (57.75–125.25)	− 0.866	0.386
CD3^+^, median (IQR),%	67.31 (54.31–69.75)	54.72 (40.50–70.09)	− 2.191	0.028
CD4^+^, median (IQR),%	40.69 (26.76–49.50)	33.60 (22.43–39.50)	− 2.293	0.022
CD8^+^, median (IQR),%	21.88 (17.25–26.61)	19.91 (14.78–26.61)	− 1.479	0.139
CD19^+^, median (IQR),%	16.82 (12.84–23.31)	19.32 (11.66–26.93)	− 1.478	0.139
NK, median (IQR),%	16.25 (8.95–24.06)	19.40 (10.79–29.06)	2.293	0.022
CD4^+^/CD8^+^, median (IQR)	1.90 (1.14–2.67)	1.74 (1.09–1.92)	− 2.191	0.028
*Cytokine profiles*				
IL-4, median (IQR), pg/mL	1.42 (1.23–2.21)	1.81 (1.34–2.43)	1.992	0.046
IL-6, median (IQR), pg/mL	19.01 (13.26–57.87)	16.87 (4.13–69.75)	− 0.314	0.753
IL-10, median (IQR), pg/mL	3.69 (3.12–4.81)	3.63 (2.75–6.00)	− 0.734	0.463
IL-17, median (IQR), pg/mL	1.17 (0.95–1.39)	1.24 (1.03–1.53)	1.572	0.116
TNF-α, median (IQR), pg/mL	2.89 (1.22–5.93)	3.75 (2.66–5.66)	0.943	0.345
IFN-γ, median (IQR), pg/mL	3.37 (2.08–11.80)	4.12 (2.08–10.70)	0.943	0.345

*ICU* intensive care unit, *NK* natural killer, *IL* interleukin, *TNF* tumor necrosis factor, *IFN* interferon

## Discussion

COVID-19 is a novel infectious disease that has led to a worldwide pandemic. According to the report from the Chinese Centers for Disease Control and Prevention, the mortality rate of COVID-19 is 2.3% [8]; however, this figure increased to 49.0% among critical cases [8]. Thus, it is of great significance to study the laboratory data and clinical development of the disease to guide management.

In this study, the clinical manifestations and laboratory data of ICU and non-ICU patients with COVID-19 were compared. In addition, the characteristics of lymphocyte subsets and cytokine profiles of peripheral blood in the enrolled patients were analyzed. It was found that most of ICU patients were older than non-ICU patients. In addition, ICU group had more patients with chronic medical diseases than non-ICU group; this indicates that older patients, in particular those with chronic medical diseases, such as hypertension and diabetes mellitus, may be more likely to develop severe COVID-19. These findings are consistent with several previous studies [12, 18, 19]. Fever, cough, and expectoration were found to be the most common symptoms. However, the above symptoms did not appear in some patients. In addition, some patients only had symptoms in the digestive system or nervous system.

Cellular immunity is an important part of the human immune system in a viral infection. Increasing evidence suggests that lymphocytes play a crucial role in airway diseases [20, 21]. Marked lymphocytopenia occurred in most patients during the acute phase of SARS and Middle East respiratory syndrome (MERS), with CD4^+^ and CD8^+^ T cells particularly affected. In addition, the degree of decrease in the T lymphocytes was associated with disease severity [22–24]. However, the mechanisms by which the viruses cause lymphocyte changes are different. In COVID-19, a growing number of studies have found that lymphocytopenia, particularly in T lymphocyte subsets, is common, especially in severe/critical cases [12, 16, 18, 25, 26], while results concerning CD19^+^ B and NK cells are inconsistent [27]. In this study, lymphocytopenia occurred in 96.2% of ICU patients and in 84.5% of non-ICU patients. Specifically, the number of CD3^+^ T cells, CD4^+^ T cells, and CD8^+^ T cells was significantly lower in ICU group than non-ICU group. While CD8^+^ T cells are vital for the elimination of virus-infected cells as a result of the secretion of perforins, granzymes, and interferons, CD4^+^ T cells participate via co-stimulating CD8^+^ T cells and CD19^+^ B cells [27]. CD4^+^ T and CD8^+^ T cells have been reported as powerful predictors of COVID-19 severity and clinical outcome in different studies, respectively [13, 15, 28]; however, there were no significant differences between the two T lymphocyte subsets in our study. The CD4^+^/CD8^+^ T cell ratio in non-ICU group and ICU group were similar, which may indicate that CD4^+^ T and CD8^+^ T cells were equally reduced in both groups [16]. CD3^+^ T cells are composed of CD4 ^+^ and CD8^+^ T cells, and CD3^+^ T cells may be a more reasonable and valuable parameter for severe disease and death. Differences in immune profiles can help to better understand the pathogenesis and clinical expression of COVID-19 [27]. Currently, the pathophysiological mechanism of lymphocyte reduction in patients with COVID-19 remains unclear and further investigations are required.

Early studies have documented that cytokine storms, also known as inflammatory storms, have occurred in a large number of patients with COVID-19. In patients with SARS, an increased number of proinflammatory cytokines in the serum, such as IL-1β, IL-6, IL-12, IFN-γ, IFN-γ-inducible protein-10, and C–C motif chemokine ligand 2, was observed and was considered to be related to pulmonary inflammation, extensive lung damage, and even multiple organ failure [29]. A previous study showed that patients with MERS also had increased concentrations of proinflammatory cytokines (IFN-γ, TNF-α, IL-15, and IL-17) [30]. Recent data have indicated that patients with COVID-19 also had high concentrations of serum cytokine profiles, such as TNF-α, IL-1, IL-6, and IFN-γ [12, 18]. In clinical work, it was found that the course of disease and lung lesions progressed rapidly, and that multiple organ failure developed over a short time in some patients with a high concentration of cytokines. In this study, it was noted that patients typically had increased concentrations of serum IL-6. Moreover, the serum IL-6 concentration was significantly higher in ICU patients than in non-ICU patients, which is in agreement with the concept of a cytokine storm [31]. However, IL-4, IL-10, IL-17, TNF-α, and IFN-γ were all nearly in the normal range. Although the exact mechanism of changes in cytokines remains to be elucidated, a higher concentration of serum cytokines seems to be associated with poor outcomes. Therefore, monitoring the changes in cytokines is of a certain significance for early detection and management of critically ill patients.

For patients with COVID-19, it is important to determine who has inherent susceptibility to develop severe or even critical disease. Monitoring COVID-19 severity is helpful in clinical decision making [32]. Early screening of critically ill patients may improve clinical outcomes. Searching for potential predictors of the severity of disease and disease outcome could help us to identify patients requiring special care, i.e., early ICU admission, intensive monitoring, and more aggressive therapy [27].

In this study, the clinical and laboratory features of patients with COVID-19 were explored. The enrolled patients were divided into two cohorts based on disease severity. Baseline characteristics, clinical presentation, and laboratory data were compared between ICU and non-ICU groups. Multivariate logistic regression analysis and ROC curve analysis were further performed. In addition, AUC and cutoff values were calculated. It was found that age, diabetes mellitus, CD3^+^ T cells < 510.50 × 10^6^/L on hospital admission, and IL-6 > 6.58 pg/mL on hospital admission were the predictive factors for the development of severe disease. ROC curve analysis showed that the AUC of CD3^+^ T cells and that of IL-6 were 0.806 and 0.785, respectively, and the AUC of combined predictor (combining age, diabetes mellitus, CD3^+^ T cell count reduction, and IL-6 elevation) was 0.887.

Among the 53 patients in ICU group, 33 patients were admitted straight to ICU due to the severity of their conditions, while the remaining 20 patients were admitted to general isolation ward and then transferred to ICU following deterioration of the disease. We found that there was no significant difference in any of the indicators of lymphocyte subsets and cytokines between direct-ICU group and late-ICU group on hospital admission. For late-ICU group, there was no significant difference in the lymphocyte subsets and cytokines from hospital admission to ICU admission, but the lymphocyte subsets showed a downward trend with disease progression. This finding also confirmed the value of lymphocyte subsets and cytokines in predicting severe illness on hospital admission. We also compared the changes in lymphocyte subsets and cytokines on hospital admission and ICU admission between severe and critical patients, and found no significant difference in all indicators.

In summary, the characteristics of lymphocyte subsets and cytokine profiles between ICU and non-ICU patients with COVID-19 were compared in this study. As a result, predictive factors for patients developing a severe condition were identified. This is helpful to identify high-risk patients as early as possible, which allows intensive monitoring and treatment to be provided at an early stage, and ultimately reduces the mortality associated with COVID-19.

This study has several potential limitations. First, the retrospective single-center design leads to missing data and unavoidable biases. However, the researchers responsible for data collection were trained before the study began so that they could correctly fill out the case report forms and reduce errors. In addition, two researchers collected data independently and checked each other’s forms for mistakes so as to minimize the bias as much as possible. Second, data were not collected continuously during the patients’ hospitalization, and, consequently, the trend of these clinical and laboratory indicators could not be described. Fortunately, all of the data were recorded in our electronic medical record system, and we plan to extract and collect parameters required for further study in the future. Third, any mutation analysis was not performed for each patient. For the studied period of time, it could be assumed that all the studied patients were infected with WT strain. It is necessary to further explore the characteristics of lymphocyte subsets and cytokines of COVID-19 patients with the variant strain.

## Conclusions

The counts of lymphocyte subsets decreased and the level of IL-6 increased in ICU COVID-19 patients compared with non-ICU COVID-19 patients. CD3^+^ T cells and IL-6 in peripheral blood may serve as independent predictors of the early identification of severe patients with WT SARS-CoV-2.

## Data Availability

All the data of this study is available on request from corresponding author.

## Abbreviations

APTT: Activated partial thromboplastin time
AUC: Area under curve
CK: Creatine kinase
CKMB: Creatine kinase-MB
COVID-19: Coronavirus disease
CRP: C-reactive protein
DBil: Direct bilirubin
HbA1c: Glycated hemoglobin A1c
ICU: Intensive care unit
IFN-γ: Interferon-γ
IL: Interleukin
IQR: Interquartile range
MERS: Middle East respiratory syndrome
NLR: Neutrophil-to-lymphocyte ratio
PCT: Procalcitonin
PT: Prothrombin time
ROC: Receiver operating characteristic curve
RT-PCR: Reverse transcription-polymerase chain reaction
SARS: Severe acute respiratory syndrome
SARS-CoV-2: Severe acute respiratory syndrome-coronavirus 2
TBil: Total bilirubin
TNF-α: Tumor necrosis factor-α
